# Racialization processes and depressive symptoms among pregnant Mexican‐origin immigrant women

**DOI:** 10.1002/ajcp.12755

**Published:** 2024-05-07

**Authors:** Alana M. W. LeBrón, Victoria E. Rodriguez, Brandy R. Sinco, Cleopatra H. Caldwell, Edith C. Kieffer

**Affiliations:** ^1^ Department of Health, Society, and Behavior, Program in Public Health University of California, Irvine Irvine California USA; ^2^ Department of Chicano/Latino Studies, School of Social Sciences University of California, Irvine Irvine California USA; ^3^ School of Medicine University of Michigan Ann Arbor Michigan USA; ^4^ Department of Health Behavior and Health Education, School of Public Health University of Michigan Ann Arbor Michigan USA; ^5^ School of Social Work University of Michigan Ann Arbor Michigan USA

**Keywords:** depressive symptoms, discrimination, immigrant, John Henryism, Latina, Mexican, pregnancy, prenatal depression

## Abstract

This study examines how racialization processes (conceptualized as multilevel and dynamic processes) shape prenatal mental health by testing the association of discrimination and the John Henryism hypothesis on depressive symptoms for pregnant Mexican‐origin immigrant women. We analyzed baseline data (*n* = 218) from a healthy lifestyle intervention for pregnant Latinas in Detroit, Michigan. Using separate multiple linear regression models, we examined the independent and joint associations of discrimination and John Henryism with depressive symptoms and effect modification by socioeconomic position. Discrimination was positively associated with depressive symptoms (*β* = 2.84; *p* < .001) when adjusting for covariates. This association did not vary by socioeconomic position. Women primarily attributed discrimination to language use, racial background, and nativity. We did not find support for the John Henryism hypothesis, meaning that the hypothesized association between John Henryism and depressive symptoms did not vary by socioeconomic position. Examinations of joint associations of discrimination and John Henryism on depressive symptoms indicate a positive association between discrimination and depressive symptoms (*β* = 2.81; *p* < .001) and no association of John Henryism and depressive symptoms (*β *= −0.83; *p* > .05). Results suggest complex pathways by which racialization processes affect health and highlight the importance of considering experiences of race, class, and gender within racialization processes.

## Highlights

- Racialization processes shape the lived experiences and health of Latiné communities.
- Latiné communities experience a high prevalence of mental health issues.
- This study focuses on racialization and depressive symptoms for Mexican‐origin immigrant women.
- Discrimination is positively associated with prenatal depressive symptoms.
- We did not find support for the John Henryism hypothesis.

## BACKGROUND

The Centers for Disease Control and Prevention has declared racism as a public health threat (Centers for Disease Control and Prevention, 2021). Racialization refers to social processes that create racial/ethnic categories and how socially constructed meanings associated with racial/ethnic groups are used to structure access to power, privileges, and health‐promoting resources (Almaguer, 2009; Omi & Winant, 2015). Racialization processes vary over time, context, and population and involve not only discrimination but also affected community members' responses to and strategies to cope with oppressive contexts (Almaguer, 2009; Ford & Airhihenbuwa, 2010; Omi & Winant, 2015; Schwalbe et al., 2000). Scholars have increasingly called for deepening understanding of the linkages between systems of racial oppression and the health of Latiné communities (a gender‐inclusive, pan‐ethnic term frequently used by Spanish‐speaking communities to refer broadly to Latina/o/x communities); variations within and across affected communities; and factors that protect the health of racially minoritized communities in the United States (Ford & Airhihenbuwa, 2010; LeBrón & Viruell‐Fuentes, 2019; Michener & LeBrón, 2022; Viruell‐Fuentes et al., 2012; Wallace et al., 2019). As racialization processes are rooted in societal processes (e.g., values, discourse), policy, and practice, these processes trickle down to affect community, family, and individual‐level lived experiences and health (Ford & Airhihenbuwa, 2010; Gee & Ford, 2011; Gee & Payne‐Sturges, 2004; Williams & Mohammed, 2009, 2013).

Nationally, an estimated 9% of Latiné and Black US adults have high levels of depressive symptoms, compared to about 7.5% of non‐Latiné white adults (Ettman et al., 2020). Other studies report that the prevalence of reported depressive symptoms among Latiné (40%) adults is approximately 1.5 times that for white adults (25.3%) (McKnight‐Eily et al., 2021). Generally, across racial/ethnic groups, the prevalence of elevated depressive symptoms among women is nearly double that for men (Ettman et al., 2020). Furthermore, an estimated 17% of pregnant women experience depression during pregnancy (Gavin et al., 2009; Underwood et al., 2016). Studies have found a higher prevalence of prenatal depression among Latinas relative to white women (Mukherjee et al., 2016).

The sociopsychobiological framework (Chae et al., 2011) posits that racism (rather than race) is a critical lens through which we should understand and address racial inequities in health. This framework outlines processes through which systems of racial oppression may become embodied to affect psychological well‐being, behavioral health, and biological risk, including historical processes related to systems of oppression, institutional inequities, personally mediated racism, and internalized racism (Chae et al., 2011). An important literature documents the intergenerational risks and spillover of discrimination during pregnancy to adverse social‐emotional outcomes for infants, identifying depressive symptoms as a mediating factor in these patterns and highlighting another pathway by which racial oppression may affect health (Liu et al., 2022; Rosenthal et al., 2018). LeBrón and Viruell‐Fuentes (2019, 2019) call for scholarship that situates the health of Latiné communities within complex and dynamic social, political, and spatial contexts within which racialization processes unfold and evolve. These patterns of high depressive symptoms among Latiné adults highlight the importance of understanding the contributions of racialization‐related stressors and responses to those stressors to the health of pregnant Latinas given the burden of depressive symptoms on this population. This paper seeks to examine the mental health implications of experiences with discrimination and John Henryism, or high effort coping in the context of social and economic oppression for pregnant Mexican‐origin immigrant women. We conceptualize discrimination and John Henryism as components of multilevel racialization processes (e.g., systemic, structural, institutional, interpersonal) and assess discrimination and John Henryism by participants' self‐report.

### Theoretical/conceptual framework

Public Health Critical Race praxis posits that the study of racial health inequities must be grounded in an understanding of the contemporary salience of racialization processes at societal, community, and individual levels, as well as an understanding of how racialization processes unfold for a given population at a given moment in time (Ford & Airhihenbuwa, 2010). In the 21st century, structural racism maintains a stronghold through, for example, racially inequitable policies, systems, and institutions (Bailey et al., 2017; Gee & Ford, 2011; LeBrón & Viruell‐Fuentes, 2019; Michener & LeBrón, 2022; Viruell‐Fuentes et al., 2012; Williams & Mohammed, 2013). In the case of the experiences of Mexican‐origin communities, the 21st century is characterized by several examples of overt and acute racism, such as the denial of driver's licenses to residents who cannot prove their authorized US citizenship (LeBrón, Schulz, et al., 2018; LeBrón, Lopez, et al., 2018), growth of immigration enforcement apparatuses (Kline, 2019; Nichols et al., 2018; Pedraza et al., 2017), immigration raids (Lopez et al., 2022; Novak et al., 2017; Lopez, 2019), and immigrant detention systems (Fleming & LeBrón, 2020). There are also many instances of more subtle forms of “the ordinariness of racism” (Ford & Airhihenbuwa, 2010, p. 1395) that can occur in day‐to‐day interactions for Mexican‐origin communities, such as unfair treatment and questioning one's legal status (LeBrón, Spencer, et al., 2018; LeBrón, Schulz, et al., 2018; LeBrón, et al., 2020; LeBrón & Viruell‐Fuentes, 2019; Rodriguez et al., 2023).

A well‐established literature links stressful life circumstances with adverse mental health outcomes, and race‐related stressors with persistent and widening racial/ethnic mental health inequities (Paradies et al., 2015). Pregnancy is a particularly sensitive time in terms of risk of discriminatory experiences (e.g., increased interactions with institutions, racialized discourse regarding pregnancy). The physiologic toll of racism over the life course and acute experiences of racism during pregnancy are well documented, particularly regarding adverse birth outcomes (Altman et al., 2019; Chae et al., 2018; Chavez, 2013; Lauderdale, 2006; Mays et al., 2007; Novak et al., 2017; Sealy‐Jefferson et al., 2020). Studies have linked discrimination with depressive symptoms in pregnancy for Latinas (Bennett et al., 2010; Harris et al., 2022; Reid et al., 2016). Depression during pregnancy is associated with poor maternal, birth, and child health outcomes (Accortt et al., 2015; Dunkel Schetter & Tanner, 2012; Kornfield et al., 2021).

Following Public Health Critical Race praxis, discrimination is conceptualized as a component of racialization processes that contributes to racial health inequities (Ford & Airhihenbuwa, 2010). The construct of discrimination has been captured by several measures of acute and ordinary forms of racism (Krieger et al., 2005; Paradies et al., 2015; Viruell‐Fuentes et al., 2011; Williams & Mohammed, 2013). Racialization processes may affect the experiences, opportunities, and health of low‐income racially minoritized communities through explicit racialization processes, such as discrimination, and through the impact of racial oppression on social and economic opportunities and access to health‐promoting resources (Ford & Airhihenbuwa, 2010). The John Henryism hypothesis has been a related lens by which we conceptualize and understand the linkages between ongoing systems of oppression, coping strategies, and health for racially minoritized populations (James et al., 1983, 1987; James, 1994). The John Henryism hypothesis is inherently an intersectional theory that captures how racialization processes intersect with socioeconomic opportunities and implications for adverse health consequences. John Henryism is coined for the famous Black folk hero, John Henry, a steel driver who competed against a mechanical steam drill to lay steel railroad tracks (James et al., 1983). Though John Henry was able to harness his strength to beat the machine, he died shortly after the steel driving contest due to mental and physical exhaustion (James et al., 1983; James, 1994). James et al. (1983) posited that the construct of John Henryism (i.e., high effort coping) captures the extent to which racially minoritized individuals—particularly Black adults—exert high effort in the context of social and economic disadvantage. Taken together, James et al. (1983) posited that those with above‐average levels of John Henryism and of lower socioeconomic position would experience adverse health outcomes. Per the John Henryism hypothesis, the combination of perceiving that one can exert high effort to control their environment and overcome structural impediments and stressors through active coping strategies, alongside racial and economic oppression, is detrimental for health (James et al., 1983). While assessed through self‐report, tests of the John Henryism hypothesis capture responses to racialization processes that are rooted in systemic, structural, and institutional processes (James et al., 1983).

First developed to understand socioeconomic variations in cardiovascular health outcomes for Black Americans, the John Henryism hypothesis has been expanded to understand mental health patterns. Some studies of the association of John Henryism—or high effort coping within the context of social and economic disadvantage—with mental health have found that high levels of John Henryism are linked with lower levels of psychological distress and psychiatric disorders and found no interaction effect of John Henryism and socioeconomic position on these mental health outcomes (Kiecolt et al., 2009), while other studies have linked higher levels of John Henryism with worse mental health outcomes (Bronder et al., 2014; Hudson et al., 2016; Perez et al., 2022). Research on the health implications of John Henryism—or high effort coping in the context of social and economic disadvantage—is most established when focusing on the health of Black adults (Hudson et al., 2016; James et al., 1983; James et al., 1992; Neighbors et al., 2007; Subramanyam et al., 2013). A smaller body of scholarship has examined the John Henryism hypothesis for Latiné communities (Kiecolt et al., 2009; LeBrón et al., 2015). Few studies have examined John Henryism through a lens that considers the health implications of John Henryism by integrating the social constructs of race, class, and gender, with a particular focus on pregnant Mexican‐origin immigrant women.

Building on Public Health Critical Race Praxis, this study seeks to deepen hypothesis generation about the relations between two constructs linked with racial health inequities: discrimination and John Henryism, and considers both unique and overlapping influences of these constructs on prenatal depressive symptoms. This study investigated racialization‐related stressors and their associations with mental well‐being for pregnant Mexican‐origin immigrant women in Detroit, Michigan. First, we examined the association between discrimination and depressive symptoms and assessed effect modification of this hypothesized association by socioeconomic position. Second, we tested the John Henryism hypothesis by examining the association between John Henryism and depressive symptoms and potential effect modification by socioeconomic position. Third, as the John Henryism hypothesis and experiences of discrimination are conceptualized as components of racialization processes, we also examined the associations of these racialization‐related stressors with depressive symptoms when accounting for both John Henrysim and discrimination simultaneously. As studies have found that reports of discrimination vary by nativity and length of US residence (for immigrants) (LeBrón et al., 2017; Viruell‐Fuentes, 2007), we also examined whether these hypothesized associations varied by length of US residence. We hypothesized that there would be a stronger association between discrimination and depressive symptoms and John Henryism and depressive symptoms for women with a longer duration of residence in the United States.

## METHODS

### Setting and community‐academic partnership

This study uses data collected at baseline from the Healthy Mothers on the Move (Healthy MOMs) randomized controlled study conducted in 2004–2006 in Southwest Detroit (Kieffer et al., 2013, 2014), a predominantly Latiné, immigrant, and low‐income community (Cruz, 2014; Data Driven Detroit, 2012). Along with the rest of the city of Detroit, at the time of this study, Southwest Detroit had experienced significant social and economic disinvestment and race‐based residential segregation (Sugrue, 1996; Williams & Collins, 2001). This intervention was developed and implemented through a partnership among representatives of the university‐based research team, a community health center that serves residents of Southwest Detroit, several other community‐based organizations, and community resident pregnant and postpartum women (Kieffer et al., 2013).

### Participants and data collection

The Healthy MOMs interventional study involved a culturally and linguistically tailored approach to leveraging a community health worker model that integrates social support and healthy lifestyle education to reduce risk factors for type 2 diabetes among pregnant and postpartum Latinas (Kieffer et al., 2013, 2014). Participants had significant improvements in dietary outcomes (Kieffer et al., 2014) and significant reductions in depressive symptoms (Kieffer et al., 2013). Participants were recruited through partner community‐based organizations; the community health center; Supplemental Nutrition Program for Women, Infants, and Children (WIC) clinics; and posters and flyers distributed in public spaces in Southwest Detroit. Eligibility criteria included being <20 weeks pregnant at the time of intervention eligibility screening—given the intervention study's focus on early intervention for diabetes prevention—identifying as Latina, living in Southwest Detroit, and being 18 years of age or older. This analysis focuses on baseline data from Mexican‐origin immigrant women who participated in the healthy lifestyle intervention or pregnancy education (control) groups, who comprised the majority of the Healthy MOMs participants (88.5%), resulting in an analytic sample of 218 pregnant participants (out of 275) at baseline. This intervention study was approved by the Institutional Review Board.

### Measures

#### Depressive symptoms

The 11‐item Center for Epidemiologic Studies Depressions Scale (CES‐D) assessed depressive symptoms over the past week (Kieffer et al., 2013; Kohout et al., 1993). Example CES‐D items include feeling depressed, feeling sad, being happy, and enjoying life. Responses ranged from never (1) to always (5). Due to small cell sizes, we collapsed responses into three categories: never or hardly ever (1), sometimes (2), and often or always (3). The depressive symptoms score was calculated as the sum of the 11 items (Cronbach's *α* = .79). Scoring of positive items (e.g., happy, enjoying life) were reverse coded. In this sample, the cumulative score ranged from 0 to 18, with higher scores indicating higher levels of depressive symptoms. The 11‐item CES‐D has been validated against the 20‐item CES‐D and has been used in the Healthy MOMs study and in other studies that involve Mexican immigrant populations (Kieffer et al., 2013).

#### Discrimination

Discrimination was measured with four items from the Everyday Discrimination Scale, which captures experiences of unfair treatment in day‐to‐day life (Williams et al., 1997). Items include frequency of being treated with less courtesy or respect than other people, receiving poorer service than other people at restaurants or stores, people acting as if they think you are not smart, and experiences of being threatened or harassed. Responses options ranged from never (1) to always (5). The discrimination score was calculated as the mean of the four items (Cronbach's *α* = .59). In this sample, the mean score ranged from 1 to 4, with higher scores indicating higher levels of discrimination. Participants who indicated discrimination happening “sometimes” or “always” for at least one of the four discrimination items were asked, “What do you think was the main reason for these experiences.” Response options included your ancestry or national origin, because you are a woman, your race, your age, your weight, your shade of skin color, your English language skills, your income or social class, because you were not born in the United States, because you live in Detroit, and other.

#### John Henryism

John Henryism was measured using the 12‐item John Henryism Active Coping Scale (James et al., 1983), which captures active, effortful coping and endorsement of narratives related to hard work and single‐minded determination to succeed within a context of social and economic disadvantage, reflecting the conceptualization of this measure as a construct that captures individual‐level strategies to respond to systemic, structural, and institutional forms of oppression. James et al. (1983) explain John Henryism as “an individual's self‐perception that [they] can meet the demands of [their] environment through hard work and determination” (p. 263). Example items include: “Hard work has really helped me to get ahead in life” and “In the past, even when things got really tough, I never lost sight of my goals.” Response options ranged from completely true (1) to completely false (4). Responses were reverse‐coded, and John Henryism was calculated as the mean of 12 items with a high score indicating high John Henryism (Chronbach's *α* = .78). Consistent with James and colleagues (James et al., 1983, 1987, 1992; Subramanyam et al., 2013), we created a dichotomous variable classifying participants with John Henryism scores above the median (3.3) as having above average John Henryism, and those at or below the median as having average or below average levels of John Henryism. The correlation coefficient for John Henryism and discrimination scales was −0.18, indicating a weak inverse correlation.

#### Moderator variables

Consistent with the John Henryism hypothesis, which contextualizes the health consequences of active, effortful coping within the socioeconomic conditions that participants are navigating, we examined two indicators of socioeconomic position: household income and educational attainment. Household income was categorized into three categories: <$20,000 (referent), $20,000 or higher, and “don't know,” based on the income distribution in the sample. Educational attainment was dichotomized to at least a high school education/general educational development (GED) and less than a high school education (referent). In post hoc analyses, we included length of US residence (continuous) as a moderator variable.

#### Covariates

Covariates included self‐reported age, gestational age, being a patient of the community health center who was a research partner, and length of residence in the United States. Age, measured in years, was categorized into four groups based on the age distribution in the sample (<23 years, 23–26 years, 27–30 years, and 31 years or higher). Gestational age at baseline was treated as a continuous variable and was derived from women's self‐reported last menstrual period. Receipt of health care at the local community health center partner was dichotomized (yes = 1; no = 0). Length of residence in the United States was derived by subtracting the self‐reported age of arrival to the United States from the participant's age at baseline. Number of live births was categorized into three groups: zero live births, one live birth, and two or more live births. Past pregnancies not carried to term were dichotomized into: no past pregnancies not carried to (0) term and at least one pregnancy not carried to term (1).

### Analysis

We used exploratory data analysis techniques to assess the frequencies and distribution of the variables and to investigate multicollinearity and assessed the correlation between the John Henryism and discrimination scales. Multivariable linear regression was used to test the first hypothesis, that higher levels of discrimination are associated with higher levels of prenatal depressive symptoms. First, we regressed prenatal depressive symptoms on discrimination, controlling for socioeconomic position and other covariates. Then, to examine potential effect modification, we included an interaction of discrimination and socioeconomic position. Interaction terms for discrimination and household income, and discrimination and educational attainment, were included in separate models. Assessments of effect modification were informed by a review of coefficients and p‐values for the interaction term and main effect variables and visual depiction of the data via scatterplots.

The second set of models examined the John Henryism hypothesis, with a focus on prenatal depressive symptoms. First, we examined whether higher levels of John Henryism, or active coping, are associated with higher levels of prenatal depressive symptoms, after adjusting for socioeconomic position and covariates. Consistent with the literature on the John Henryism hypothesis, to test for the second hypothesis, that higher levels of John Henryism are associated with higher levels of prenatal depressive symptoms in the context of socioeconomic disadvantage, we included in the models an interaction term for John Henryism and indicators of socioeconomic position. Interaction terms for John Henryism and household income, and John Henryism and educational attainment, were included in separate models. Finally, to assess the health implications of active coping and discrimination together, in the same model, we regressed prenatal depressive symptoms on John Henryism and discrimination. Finally, each model included an assessment of goodness‐of‐fit statistics (Akaike and Bayesian information criteria [AIC, BIC], *R* ^2^, and adjusted *R* ^2^). The goodness of fit of the models was evaluated by comparing Akaike information criteria (AIC), Bayesian information criteria (BIC), and adjusted *R* ^2^. Within each of Tables 3, 4, 5, the best‐fitting model had the lowest AIC and BIC, along with the highest adjusted *R* ^2^. We considered adding variables to each model if they were statistically significant, in addition to improving AIC, BIC, and adjusted *R* ^2^.

## RESULTS

Demographic characteristics of the analytic sample (*n* = 218) are presented in Table 1. Among this sample of pregnant Mexican‐origin immigrant women, nearly one‐third (30.7%) were between 23 and 26 years old, followed by 31 years and older (28.0%), 27–30 years (21.6%) and less than 23 years (19.7%). The mean gestational age at baseline was 17.4 weeks (standard deviation [SD] = 4.4; range = 7–27). About half (53.2%) of participants had a self‐reported household income of less than $20,000. Approximately half (53.7%) of participants had less than a high school education. The majority (67.4%) of participants received health care from the partner local community health center. The mean length of US residence among Mexican‐origin immigrant women in this sample was 6.1 years (SD = 4.8; range = <1–36 years). About one‐ quarter (26.2%) of participants reported not having a previous live birth, while 31.2% reported one past live birth, and 42.7% reported having two or more prior live births. The majority of participants (96.3%) with at least one prior pregnancy reported having at least one past pregnancy not carried to term. The median John Henryism score was 3.3 (SD = 0.4; range = 1.9–4), which is slightly below that reported in another study involving US‐born and immigrant Latinés (LeBrón et al., 2015). The mean discrimination score was 1.8 (SD = 0.6; range = 1–4). The mean baseline CES‐D depressive symptoms score in this sample was 6.8 (SD = 3.9; range = 0–18).

**Table 1:** Descriptive statistics, healthy Mothers on the Move study, 2004–2006 (*n* = 218).

	Total sample				Discrimination^a^						John Henryism^b^			
	(*n* = 218)				Low (*n* = 129)			High (*n* = 89)			Low (*n* = 117)		High (*n* = 101)	
	%	Mean (SD)	Median (IQR)	Range	%	Mean (SD)	Median (IQR)	%	Mean (SD)	Median (IQR)	%	Mean (SD)	%	Mean (SD)
Age														
<23 years	19.7				14.0			28.1			25.6		12.9	
23–26 years	30.7				34.9			24.7			32.5		28.7	
27–30 years	21.6				19.4			24.7			22.2		20.8	
31+ years	28.0				31.8			22.5			19.7		37.6	
Gestational age		17.44 (4.35)		7–27		17.44 (4.38)			17.43 (4.32)			17.15 (4.45)		17.76 (4.22)
Household income														
<$20,000	53.2				50.4			57.3			53.0		53.5	
$20,000 or higher	17.4				15.5			20.2			18.0		16.8	
Don't know	29.4				34.1			22.5			29.1		29.7	
Educational attainment														
Less than high school education	53.7				54.3			52.8			49.6		58.4	
High school education/GED or higher	46.3				45.7			47.2			50.4		41.6	
Receive health care from local community health left	67.4				68.2			66.3			68.4		66.3	
Length of Residence in the United States (years)		6.06 (4.84)		0–36		6.10 (5.22)			6.01 (4.27)			5.74 (4.54)		6.45 (5.17)
Previous live births														
0	26.2				24.0			29.2			30.8		20.8	
1	31.2				34.1			27.0			33.3		28.7	
2+	42.7				41.9			43.8			35.9		50.5	
Past pregnancies not carried to term^c^														
No past pregnancies	3.7				2.3			5.6			4.3		3.0	
At least one past pregnancy	96.3				97.7			94.4			95.7		97.0	
John Henryism														
Continuous scale		3.28 (0.44)	3.33 (2.92, 3.67)	1.92–4		3.34 (0.42)	3.34 (3.08, 3.67)		3.19 (0.45)	3.20 (2.83, 3.58)				
Discrimination		1.76 (0.58)		1–4								1.80 (0.60)		1.72 (0.57)
Depressive symptoms		6.77 (3.94)		0–18		5.77 (3.60)			8.22 (3.97)			7.24 (3.93)		6.23 (3.90)

Abbreviations: GED, general educational development; IQR, interquartile range; SD, standard deviation.

^a^ Discrimination categories: low discrimination: 1–1.76; high discrimination: 1.77–4.

^b^ John Henryism categories: low John Henryism: 1.92–3.33; high John Henryism: 3.34–4. Median and IQR provided for John Henryism given median split of this variable, consistent with the John Henryism literature.

^c^ Among those with another prior pregnancy.

### Discrimination and prenatal depressive symptoms

Among the 52.8% of participants who reported sometimes or always experiencing discrimination, the most common attribution for their experience(s) of discrimination was because of their English language skills (46.1%), followed by their race (16.5%), ancestry or national origin (10.4%), and because they were not born in the United States (7.8%) (Table 2). Tests of the associations between discrimination and prenatal depressive symptoms are presented in Table 3. Discrimination was positively and significantly associated with prenatal depressive symptoms (Model 1; *β* = 2.84; 95% confidence interval [CI]: 2.00, 3.69, *p* < .001) when adjusting for covariates. The association between discrimination and prenatal depressive symptoms was not significantly moderated by household income (Model 2; *β* = −1.36; 95% CI: −4.09, 1.36, *p* > .05) nor by educational attainment (Model 3; *β* = −1.58; 95% CI: −3.22, 0.07, *p* > .05). In post hoc analyses (results not shown), given aforementioned reports of discrimination linked with language, race, ancestry or national origin, and nativity, we examined whether the association between discrimination and prenatal depressive symptoms was modified by length of US residence and did not find support for this hypothesis.

**Table 2:** Main reason for discrimination (%), among participants reporting “sometimes” or “always” experiencing discrimination (*n* = 115).

	*n*	%
Your English language skills	53	46.1
Your race	19	16.5
Your ancestry or national origin	12	10.4
Because you were not born in the United States	9	7.8
Your age	5	4.4
Because you are a woman	2	1.7
Your weight	2	1.7
Your income or social class	2	1.7
Because you live in Detroit	2	1.7
Your shade of skin color	1	0.9
Other	8	7.0

**Table 3:** Depressive symptoms regressed on discrimination and socioeconomic position (*n* = 218).

	Model 1		Model 2		Model 3	
	*β*	95% CI	*β*	95% CI	*β*	95% CI
*Main independent variable*						
Discrimination	2.84	(2.00, 3.69)**	3.03	(1.91, 4.15)**	3.51	(2.42, 4.61)**
*Moderators*						
Household income						
Under $20,000	Referent	—	Referent	—	Referent	—
$20,000 or higher	−0.86	(−2.21, 0.48)	1.63	(−3.53, 6.78)	−0.87	(−2.20, 0.46)
Don't know	−0.27	(−1.40, 0.86)	−0.22	(−3.55, 3.12)	−0.23	(−1.35, 0.89)
Household income × discrimination						
Under $20,000			Referent	—		
$20,000 or higher			−1.36	(−4.09, 1.36)		
Don't know			−0.02	(−1.84, 1.79)		
Educational attainment						
<High school	Referent	—	Referent	—	Referent	—
High school grad/GED	0.28	(−0.73, 1.28)	0.28	(−0.73, 1.29)	3.03	(−0.01, 6.08)*
Educational attainment × discrimination						
<High school					Referent	—
High school grad/GED					−1.58	(−3.22, 0.07)
Model fit measures						
AIC	1182.065		1184.975		1180.265	
BIC	1229.448		1239.127		1231.033	
*R* ^2^	.246		.250		.259	
Adjusted *R* ^2^	.198		.194		.208	

*Note*: All models controlled for age, gestational age, received health care from local community health left, length of time in the United States, number of previous live births, and past pregnancies not carried to term.

Abbreviations: AIC, Akaike information criterion; BIC, Bayesian information criterion; CI, confidence interval; GED, general educational development.

* *p* < .05;

** *p* < .001 (two‐tailed tests).

### John Henryism and prenatal depressive symptoms

Results of tests of the associations between John Henryism and prenatal depressive symptoms are shown in Table 4. Model 1 indicates a statistically significant inverse association between John Henryism and prenatal depressive symptoms (Model 1; *β* = −1.57; 95% CI: −2.78, −0.36, *p* < .05) when adjusting for covariates. Associations between John Henryism and prenatal depressive symptoms were not significantly modified by household income (Model 2; *β* = 2.20; 95% CI: −1.17, 5.57, *p* > .05) nor by educational attainment (Model 3; *β* = 1.66; 95% CI: −0.76, 4.08, *p* > .05). In post hoc analyses (results not shown), we examined whether the association between John Henryism and prenatal depressive symptoms was modified by length of US residence and did not find support for this hypothesis.

**Table 4:** Depressive symptoms regressed on John Henryism and socioeconomic position (*n* = 218).

	**Model 1**		**Model 2**		**Model 3**	
	***β***	**95% CI**	***β***	**95% CI**	***β***	**95% CI**
*Main independent variable*						
John Henryism	−1.57	(−2.78, −0.36)*	−1.96	(−3.57, −0.35)*	−2.33	(−3.97, −0.69)*
*Moderators*						
Household income						
Under $20,000	Referent	—	Referent	—	Referent	—
$20,000 or higher	−0.94	(−2.40, 0.52)	−8.14	(−19.28, 3.01)	−1.08	(−2.54, 0.39)
Don't know	−0.60	(−1.82, 0.63)	−0.74	(−10.02, 8.54)	−0.60	(−1.82, 0.62)
Household income × John Henryism						
Under $20,000			Referent	—		
$20,000 or higher			2.20	(−1.17, 5.57)		
Don't know			0.04	(−2.78, 2.85)		
Educational attainment						
<High school	Referent	—	Referent	—	Referent	—
High school grad/GED	−0.05	(−1.14, 1.04)	−0.15	(−1.25, 0.95)	−5.51	(−13.52, 2.51)
Educational attainment × John Henryism						
<High school					Referent	—
High school grad/GED					1.66	(−0.76, 4.08)
Model fit measures						
AIC	1217.816		1219.927		1217.853	
BIC	1265.199		1274.079		1268.621	
*R* ^2^	.112		.119		.120	
Adjusted *R* ^2^	.055		.054		.059	

*Note*: All models controlled for age, gestational age, received health care from local community health left, length of time in the United States, number of previous live births, and past pregnancies not carried to term.

Abbreviations: AIC, Akaike information criterion; BIC, Bayesian information criterion; CI, confidence interval; GED, general educational development.

* *p* < .05.

### Discrimination, John Henryism, and prenatal depressive symptoms

**Figure 1:**
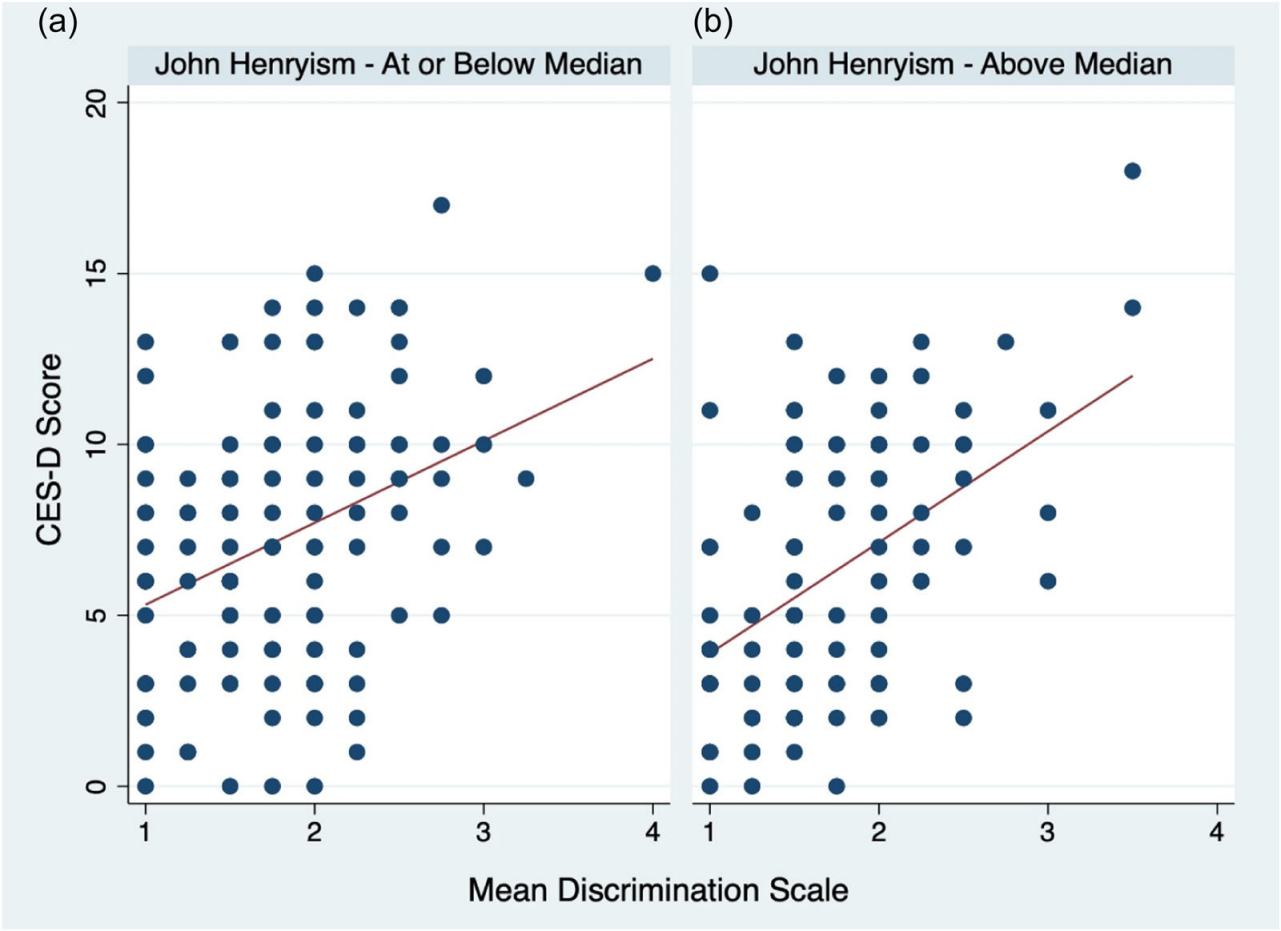
Prenatal depressive symptoms (CES‐D) and discrimination patterns, by John Henryism prenatal depressive symptoms (as measured by the CES‐D) and discrimination patterns for pregnant Mexican‐origin immigrant women with John Henryism scores at or below the median (a) and John Henryism scores above the median (b). CES‐D, Center for Epidemiologic Studies Depressions Scale.

We then examined the association of prenatal depressive symptoms with discrimination and John Henryism, including these constructs in the same model (Table 5). When John Henryism and discrimination were included as independent variables in the same model, the previously (shown in Table 4, Model 1) identified inverse and significant association of John Henryism and prenatal depressive symptoms became attenuated and did not reach statistical significance (Table 5, Model 1; *β* = −0.83; 95% CI: −1.81, 0.14, *p* > .05). Additionally, the significant positive association of higher levels of discrimination with higher levels of prenatal depressive symptoms (shown previously in Table 3, Model 1) decreased slightly in magnitude though it remained statistically significant (*β* = 2.81; 95% CI: 1.97, 3.66, *p* < .001). Tests of effect modification of the association between discrimination and prenatal depressive symptoms by John Henryism were not statistically significant (Table 5, Model 2; *β* = 1.21; 95% CI: −0.47, 2.88, *p* > .05). Presented in Figure 1 is a plot of prenatal depressive symptoms and mean discrimination scores for pregnant Mexican‐origin immigrant women for those with John Henryism scores that were at or below the median (Figure 1a) and those that were above the median (Figure 1b). As shown in these scatter plots, there was a slightly wider spread or patterning of discrimination and prenatal depressive symptoms for women who reported high levels of John Henryism (above the median of John Henryism scale).

**Table 5:** Depressive symptoms regressed on discrimination, John Henryism, and socioeconomic position (*n* = 218).

	Model 1		Model 2	
	*β*	95% CI	*β*	95% CI
*Main independent variables*				
Discrimination	2.81	(1.97, 3.66)*	2.27	(1.14, 3.40)*
John Henryism				
At or below median	Referent	—	Referent	—
Above median	−0.83	(−1.81, 0.14)	−2.95	(−6.05, 0.14)
*Moderators*				
Household income				
Under $20,000	Referent	—	Referent	—
$20,000 or higher	−0.89	(−2.23, 0.45)	−1.01	(−2.36, 0.33)
Don't know	−0.27	(−1.40, 0.85)	−0.31	(−1.43, 0.81)
Household income × discrimination				
Under $20,000				
$20,000 or higher				
Don't know				
Educational attainment				
<High school	Referent	—	Referent	—
High school grad/GED	0.24	(−0.76, 1.24)	0.26	(−0.74, 1.26)
Educational attainment × discrimination				
<High school				
High school grad/GED				
John Henryism × discrimination				
At or below median			Referent	—
Above median			1.21	(−0.47, 2.88)
Model fit measures				
AIC	1181.026		1180.854	
BIC	1231.793		1235.006	
*R* ^2^	.256		.264	
Adjusted *R* ^2^	.205		.209	

*Note*: All models controlled for age, gestational age, received health care from local community health left, length of time in the United States, number of previous live births, and past pregnancies not carried to term.

Abbreviations: AIC, Akaike information criterion; BIC, Bayesian information criterion; CI, confidence interval; GED, general educational development.

* *p* < .001 (two‐tailed tests).

## DISCUSSION

Guided by Public Health Critical Race praxis (Ford & Airhihenbuwa, 2010), this study sought to examine the unique and potentially overlapping influence of discrimination and John Henryism, or active coping in the midst of racial and economic oppression on the mental well‐being of pregnant Mexican‐origin immigrant women. There are three key findings from this study. First, higher levels of discrimination were associated with worse prenatal depressive symptoms, an association that persisted after controlling for John Henryism. Second, participant attributions of experiences of discrimination were highly racialized, linked with their language use, racial background, or nativity. Third, patterns suggested that cross‐sectionally, once models accounted for reports of discrimination, we did not find evidence to support the John Henryism hypothesis in this population of pregnant Mexican‐origin immigrant women. Below, we unpack each of these findings.

Consistent with a growing literature (LeBron et al., 2014; LeBron, Spencer, et al., 2018; LeBrón & Viruell‐Fuentes, 2019) regarding the mental well‐being of Latiné communities, we found that discrimination was linked with higher levels of prenatal depressive symptoms among pregnant Mexican‐origin immigrant women. We found no support for the hypothesis that the association of discrimination with prenatal depressive symptoms would be worsened for participants with a lower socioeconomic position. Furthermore, after adjusting for John Henryism, discrimination remained significantly associated with higher levels of prenatal depressive symptoms, suggesting that discrimination has an inimical effect on prenatal depressive symptoms, independent of John Henryism. Analysis of participants' attributions of discrimination highlights the multiple racialized layers that pregnant Mexican‐origin immigrant women encounter and the importance of considering multiple identities in racialization processes. Women's reporting of discrimination due to language use, race, and nativity align with literature documenting increases in discriminatory discourse, policy, and treatment during this study period (2004–2006), particularly racialized discourse that constructs pregnant Mexican‐origin immigrant women as a reproductive threat to the United States (Chavez, 2013; LeBrón, Schulz, et al., 2018). This period has been characterized by a growth of interior and border immigration enforcement apparatuses nationwide and in Detroit, a northern border city, which has contributed to increased immigration enforcement actions such as immigration raids and collaborations between police and immigration enforcement agencies (De Genova, 2007; Kline, 2019; Lopez, 2019; Novak et al., 2017). Past studies found that Mexican‐origin immigrant women living in Southwest Detroit primarily interacted within their ethnic enclave, with limited experiences with institutions and communities outside of their neighborhood, which in turn reduced their interactions with non‐Latines and institutional agents who may be common sources of experiences of discrimination (Viruell‐Fuentes & Schulz, 2009; Viruell‐Fuentes, 2007). Subsequent scholarship demonstrated that Mexican‐origin women in this same community (Southwest Detroit) experienced an increase in surveillance from interior and border immigration enforcement agencies in subsequent years (LeBrón, Schulz, et al., 2018; LeBrón et al., 2022). One study found that Mexican‐origin women identified language use and physical features as racializing markers that police and immigration officials leveraged in their surveillance of Latiné communities in Southwest Detroit (LeBrón, Schulz, et al., 2018). In this study, Mexican‐origin immigrant women's reports of discrimination linked with language use, race, and nativity likely intersected with concerns about immigration enforcement actions affecting their mixed‐status Latiné community, meaning that community members hold a range of legal statuses, ranging from undocumented statuses to temporary protected statuses to US citizens.

Some important characteristics of experiences with immigration enforcement apparatuses and anti‐immigrant sentiments and practices in Detroit—a northern border community—warrant discussion. In Detroit, Latiné communities—while part of the global majority—are a “minority minority.” For example, while in 2000 Latiné immigrants (of whom the majority were Mexican‐origin) comprised 43.5% of the Detroit immigrant community (U.S. Census Bureau, 2000), Latiné immigrants and their social network members are a smaller segment of racially minoritized communities in and around Detroit. This social and demographic context may contribute to smaller social networks and unique experiences with racialized illegality (García, 2017)—institutional and interpersonal processes of being racialized as having an unauthorized legal status—given smaller community sizes and deep and growing entanglement between local police and immigration authorities in Detroit, Michigan. For example, a qualitative study of Mexican‐origin women in Detroit's experiences with immigration enforcement found that in the Motor City—for which driving is essential for fulfilling caregiving, employment, and health care responsibilities—Mexican‐origin residents recalled encounters with police that quickly escalated to encounters with immigration enforcement agencies (LeBrón, Schulz, et al., 2018, Under Review). In that study, Mexican‐origin women recalled experiences of being pulled over by police in and around their predominantly Latiné neighborhood, police assuming that drivers spoke monolingual Spanish and in turn calling immigration agencies for interpretation services, and/or police assumptions of an unauthorized US presence for drivers who may have lacked a current, unexpired driver's license (racializing markers of [il]legality) (LeBrón, Schulz, et al., 2018). To inform efforts to redress discrimination, future studies should examine Mexican‐origin women's experiences of discrimination in various systems and institutions (e.g., health care, education, work); the racialization processes that underpin these experiences; and the contributions of unique social, economic, spatial, and political contexts to these processes (e.g., northern and southern border communities, nonborder communities).

This cross‐sectional study found limited support for the John Henryism hypothesis, notably that higher levels of active coping combined with low socioeconomic position are inimical for mental health, a finding that aligns with some studies of main effects of John Henryism for the mental health of Black women (Bronder et al., 2014; Perez et al., 2022). Findings from this study suggest that while John Henryism (or active coping in the context of racial and economic oppression) initially appeared protective of mental health for pregnant Mexican‐origin immigrant women, after adjusting for discrimination, experiences of discrimination appeared to dilute the health‐protective implications of John Henryism. Additionally, when explicitly incorporating a measure of racialization‐related stressors, our findings did not find support for the John Henryism hypothesis in the context of prenatal depressive symptoms for pregnant Mexican‐origin immigrant women. There are several factors related to these findings that warrant unpacking. First, the John Henryism hypothesis has been most advanced in samples that include Black adults and, in particular, Black men and have focused on cardiovascular outcomes (Hudson et al., 2016; James et al., 1983, 1992; James, 1994; Subramanyam et al., 2013). While James et al. (1983) note that the John Henryism hypothesis may not be limited to Black adults or to men, these findings suggest the importance of considering racial and gendered complexities with histories of racialization processes and implications for different health outcomes over the life course. In this sample, pregnant Mexican‐origin immigrant women had slightly lower John Henryism scores (median John Henryism score; 3.33) than identified in a multiracial study of African American and Latiné adults in Detroit (median John Henryism score: 3.5) (LeBrón et al., 2015). The formative research that informed the development of the Healthy MOMs intervention found that pregnant and postpartum Latinas in Southwest Detroit experienced social isolation and multiple stressors including those related to caregiving and family responsibilities and neighborhood safety (Kieffer et al., 2002). Furthermore, women reported few social network members beyond their male partners given that many of their kin networks were largely based in Mexico (Kieffer et al., 2002). Within this context, women may have had low expectations of control, which may have influenced the role of active coping in the context of racial and economic oppression.

Second, we do not find support for the John Henryism hypothesis in this population, based on findings indicating that higher levels of John Henryism were associated with lower prenatal depressive symptoms and that socioeconomic status did not moderate the hypothesized association of John Henryism and prenatal depressive symptoms. These findings point to the importance of considering alternative hypotheses related to the directionality of these hypothesized associations, such as the possibility that those with more favorable prenatal mental health may be more likely to endorse perspectives of motivation and determination that characterize narratives of American values (Griggs & Mallinger, 2004). Moreover, these findings suggest the importance of keeping our analytical gaze focused on structural and institutional forms of oppression that produce health inequities, and that we take care to avoid narrow interpretations of tests of the John Henryism hypothesis that may contribute to narratives that place the blame of adverse health outcomes on racially minoritized communities.

The findings indicating that length of residence in the United States did not modify the association between discrimination and mental health for this sample of pregnant Mexican‐origin immigrant women also warrants unpacking. Past scholarship has noted that recent Latiné immigrants may have a shorter period of exposure to US racialization processes and accordingly may have different experiences with, interpretations of, and naming of racialization processes (such as reporting discrimination and experiences with systems of oppression) relative to their US‐born counterparts (LeBrón et al., 2017; Viruell‐Fuentes, 2007, 2011). For example, Viruell‐Fuentes (2007) found that among Mexican‐origin women in Detroit, Michigan, first‐generation women's social lives revolved around their predominantly Latiné community, with limited interactions with institutions and out‐group members. In contrast, second‐generation Mexican‐origin women often had interactions with US institutions (e.g., education system, employers, etc.) and out‐group members (e.g., non‐Latiné communities) and as a function of their socialization in the United States during childhood, developed language and frameworks to characterize and name as discrimination their experiences with racialization processes (Viruell‐Fuentes, 2007). It is possible that similar dynamics may contribute to (a) shaping exposure to forms of interpersonal or institutional discrimination captured by the Everyday Discrimination Scale and (b) socialization and consciousness‐raising about frameworks that may in turn contribute to naming or reporting lived experiences with racialization processes as discrimination in this sample. In the present study, we found that length of US residence did not modify the association between discrimination and prenatal depressive symptoms for pregnant Mexican‐origin immigrant women. Future research is needed regarding lived experiences with racialization processes for heterogeneous Latiné immigrant communities, factors that may contribute to differential exposures to and characterization of discrimination, and health implications.

Additional factors are important to consider when interpreting these findings regarding racialization, responses to racialization, and implications for prenatal depressive symptoms. For example, other studies suggest there may be differences in how women respond to and manage stress relative to men (Perez et al., 2022). Furthermore, this study involved a sample of pregnant participants. Pregnancy is a unique moment when birthing people are encouraged to rest, surrender, and lower their stress levels, and when they may be undergoing a mindset shift to incorporate the multifaceted components of being a provider and caregiver (Kieffer et al., 2002). Family and individual transformations during pregnancy could affect the associations between John Henryism and mental health. Future studies are warranted regarding John Henryism and health across the perinatal period. For example, might one's perspective on active coping change over particular moments in the life course (e.g., childbearing)? Additionally, future scholarship should engage the environmental affordances model (Jackson et al., 2010; Mezuk et al., 2013) by examining potential short‐, intermediate‐, and long‐term effects of racialization processes on health.

### Strengths and limitations

Strengths of this study include extending tests of the John Henryism hypothesis to include pregnant Mexican‐origin immigrant women and examining the joint health implications of discrimination and John Henryism, two areas that deserve deeper attention in the study of racialization processes, intersectionality, and health. As with all studies, this study is also characterized by some limitations. First, this analysis involves a relatively modest sample size, which prohibited more sophisticated intersectional analyses. Second, this analysis is cross‐sectional, thus the health implications of discrimination and John Henryism in this study should be understood within a particular moment of time. Third, while this study set out to examine effect modification of the association of John Henryism and discrimination on health, the relatively limited socioeconomic variation in this sample may impede tests of these associations (i.e., 8.7% of participants in our sample had an educational attainment of some college, associate's degree, or a bachelor's degree). Fourth, the discrimination scale included in this study queried about recent experiences of day‐to‐day discrimination. This measure may not have captured past experiences of discrimination nor more acute experiences of racism that women may navigate in their daily lives. Relatedly, the relatively low Cronbach's *α* (.59) for the discrimination scale suggests modest internal consistency. Future studies would benefit from other analytic approaches to using this measure to assess health implications, such as focusing on number of domains of reported discrimination or an intersection of number of domains and frequency or burden. Fifth, this study drew upon baseline data at time of enrollment in the Healthy MOMs intervention study, which included pregnant Latinas who were <20 weeks gestation given the intervention study's focus on diabetes. Importantly, the current study captures the health implications of discrimination reported at baseline (first trimester and early second trimester), and thus may not capture the cumulative effects of racialization processes on prenatal depressive symptoms. Sixth, data from this study were collected in 2004–2006, which constituted a particular moment in the US racial context. In line with Public Health Critical Race praxis (Ford & Airhihenbuwa, 2010), it is important to consider racialization processes as they unfold in particular temporal and place‐based contexts (Almaguer, 2009; Omi & Winant, 2015; Schwalbe et al., 2000). In the years since the Healthy MOMs intervention, the United States has seen increased xenophobia in national and local discourse, accompanied with federal, state, and local policies that have been largely exclusionary towards Latiné and immigrant communities (De Genova, 2007; Kline, 2016; LeBrón, Schulz, et al., 2018; Lopez, 2019). The economic and health consequences of racialization processes for Latiné communities in recent years are beginning to be well‐documented (Chavez et al., 2019; Rugh & Hall, 2016). Future studies should consider this shifting racial context and revisit the associations between discrimination, John Henryism, socioeconomic position, and health involving a more recent sample of pregnant Mexican‐origin immigrant women, consider regional variations in these associations, and/or look at these associations longitudinally. Additionally, while this individual‐level empirical study of ways in which racialization processes may affect prenatal depressive symptoms for Mexican‐origin women is embedded within a critical analysis of the context of racism and colonization that may underlie health, we are cognizant that these research questions and findings may be susceptible to what Tuck (2009) frames as “damage‐centered narratives.” We recognize the nascent stage of public health scholarship seeking to understand the contributions of racial oppression and structural drivers of health for Latiné communities. We also heed Tuck's call for “desire‐based research” that explores responses to racialization processes that do not succumb to victim‐blaming, which is an important area for future research that is particularly amenable to ethnographic scholarship (Tuck, 2009).

## CONCLUSIONS

This cross‐sectional study examined the mental health implications of experiencing and navigating racialization processes for a relatively low‐income sample of pregnant Mexican‐origin immigrant women in Detroit, Michigan. Specifically, we examined the unique and joint mental health implications of discrimination and John Henryism. Results indicate that discrimination is inimical for prenatal mental health during this sensitive life stage and we did not find evidence to support the John Henryism hypothesis for this sample of pregnant Mexican‐origin immigrant women. These findings indicate that to promote the health of Latiné and immigrant communities and birthing people, we must invest in policies and programs that support their full inclusion, with particular attention to addressing and mitigating the impact of the joint forces of racism, classism, and sexism in the United States.
